# Crown-of-Thorns Sea Star Acanthaster cf. solaris Has Tissue-Characteristic Microbiomes with Potential Roles in Health and Reproduction

**DOI:** 10.1128/AEM.00181-18

**Published:** 2018-06-18

**Authors:** Lone Høj, Natalie Levy, Brett K. Baillie, Peta L. Clode, Raphael C. Strohmaier, Nachshon Siboni, Nicole S. Webster, Sven Uthicke, David G. Bourne

**Affiliations:** a Australian Institute of Marine Science, Townsville, Queensland, Australia; b AIMS@JCU, Division of Research and Innovation, James Cook University, Townsville, Queensland, Australia; c College of Science and Engineering, James Cook University, Townsville, Queensland, Australia; d Centre for Microscopy, Characterisation and Analysis, The University of Western Australia, Perth, Western Australia, Australia; e School of Biological Sciences, The University of Western Australia, Perth, Western Australia, Australia; f The Oceans Institute, The University of Western Australia, Perth, Western Australia, Australia; g Australian Centre for Ecogenomics, University of Queensland, Brisbane, Queensland, Australia; University of Manchester

**Keywords:** Mollicutes, Spiroplasma, coral reefs, dysbiosis, echinoderms, microbiome, sea stars

## Abstract

Outbreaks of coral-eating crown-of-thorns sea stars (CoTS; Acanthaster species complex) cause substantial coral loss; hence, there is considerable interest in developing prevention and control strategies. We characterized the microbiome of captive CoTS and assessed whether dysbiosis was evident in sea stars during a disease event. Most tissue types had a distinct microbiome. The exception was female gonads, in which the microbiomes were highly variable among individuals. Male gonads were dominated (>97% of reads) by a single Mollicutes-related operational taxonomic unit (OTU). Detailed phylogenetic and microscopy analysis demonstrated the presence of a novel Spiroplasma-related bacterium in the spermatogenic layer. Body wall samples had high relative abundance (43 to 64% of reads) of spirochetes, likely corresponding to subcuticular symbionts reported from many echinoderms. Tube feet were characterized by Hyphomonadaceae (24 to 55% of reads). Pyloric cecal microbiomes had high alpha diversity, comprising many taxa commonly found in gastrointestinal systems. The order Oceanospirillales (genera Endozoicomonas and Kistimonas) was detected in all tissues. A microbiome shift occurred in diseased individuals although differences between tissue types were retained. The relative abundance of spirochetes was significantly reduced in diseased individuals. Kistimonas was present in all diseased individuals and significantly associated with diseased tube feet, but its role in disease causation is unknown. While Arcobacter was significantly associated with diseased tissues and Vibrionaceae increased in diversity, no single OTU was detected in all diseased individuals, suggesting opportunistic proliferation of these taxa in this case. This study shows that CoTS have tissue-characteristic bacterial communities and identifies taxa that could play a role in reproduction and host health.

**IMPORTANCE** Coral-eating crown-of-thorns sea stars (CoTS; Acanthaster species complex) are native to the Indo-Pacific, but during periodic population outbreaks they can reach extreme densities (>1,000 starfish per hectare) and function as a pest species. On the Great Barrier Reef, Australia, CoTS have long been considered one of the major contributors to coral loss. There has been significant investment in a targeted control program using lethal injection, and there is interest in developing additional and complementary technologies that can increase culling efficiencies. The biology of CoTS has been studied extensively, but little is known about their associated microbiome. This cultivation-independent analysis of the CoTS microbiome provides a baseline for future analyses targeting the functional role of symbionts, the identification of pathogens, or the development of reproduction manipulators.

## INTRODUCTION

Crown-of thorns sea stars (CoTS; Acanthaster spp., excluding Acanthaster brevispinus) are corallivorous carnivores that display long-term boom-bust population cycles with densities reaching plague proportions. CoTS were previously thought to belong to a single species, Acanthaster planci. It is now recognized that there are at least four species in the Indo-Pacific, and the name Acanthaster solaris is proposed for the Pacific species that is native to the Great Barrier Reef (GBR) in Australia (1). Here, this species will be referred to as Acanthaster cf. solaris or crown-of-thorns starfish (CoTS). Four population outbreaks of CoTS have been documented on the GBR since the 1960s (2, 3), and it was estimated that CoTS contributed to approximately 42% of the decline in coral cover on the GBR in the period from 1985 to 2012 (4). As a consequence, local management options for CoTS have received considerable attention (5–7).

Marine invertebrates have associated microbiomes that play major roles in their biology, including settlement induction, development, metamorphosis, reproduction, digestion, and nutrition (8). Despite the critical importance of the microbiome to host health, studies of echinoderm microbiology are scarce, and most have been triggered by disease outbreaks in the wild (9, 10) or in aquaculture facilities (11, 12). Recently, however, molecular surveys of bacteria associated with healthy sea urchins (13–15), holothurians (16), and the coelomic fluid of the sea star species Patiria pectinifera and Asterias amurensis (17) were reported. Many echinoderms, including many sea stars, have subcuticular bacteria (SCBs) localized in the lumen between epidermal cells and the outer cuticle (18–22). The presence of SCBs appears to be related to host classification, in most cases at the family level (20). Although SCBs have not previously been investigated for the family Acanthasteridae, they have been detected in other members of the order Valvatida (20, 22). While the functional role of SCBs is not clear, it has been hypothesized that they can provide nutrition and antimicrobial protection (22).

To date, all studies of bacteria in CoTS have been cultivation based (23–28), biasing our understanding of their microbiome and precluding assessment of total microbial diversity in this ecologically important sea star. Sutton and Trott (23) found that seasonal factors had no effect on microbial composition in apparently healthy individuals and suggested that the most dominant bacterial type could be a specific symbiont. Vibrio, Photobacterium, and Pseudoalteromonas species have been isolated from healthy CoTS (25–28). Several potential pathogens have also been isolated from CoTS displaying disease symptoms (lesions, tissue degeneration, loss of turgor, and collapsed spines) including Vibrio spp., Pseudomonas, and Moraxella (24, 26, 27). Vibrio has been a focus of CoTS microbiology research to date, but without a culture-independent assessment of the total microbial community, it is difficult to ascertain its relative importance to host health state.

**FIG 1:**
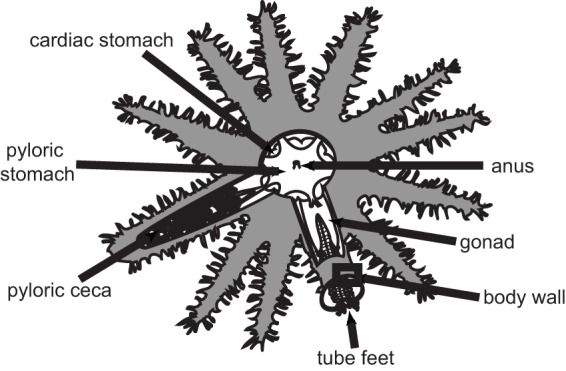
Schematic drawing of Acanthaster cf. solaris showing the location of sampled somatic tissues (body wall, tube feet, and pyloric ceca) and gonads.

There is increasing appreciation that many diseases in humans, and most likely also in marine systems, are linked to microbial imbalance (dysbiosis) or polymicrobial infections (29). This challenges the traditional approach of attempting to isolate single pathogenic agents by standard methods in order to understand and describe marine diseases and emphasizes the need to investigate the total microbiome in healthy as well as diseased individuals. The aim of the current study was to provide a microbial baseline for different Acanthaster cf. solaris tissues and determine how these change during the onset of disease. Healthy and diseased individuals were sampled from CoTS held in outdoor tanks, and the microbiomes associated with body wall, tube feet, pyloric ceca, and gonads (Fig. 1) were analyzed by amplicon sequencing of 16S rRNA genes, histology, and electron microscopy. The taxonomic position of one dominant phylotype was analyzed in more detail by cloning and Sanger sequencing of the corresponding 16S rRNA genes.

## RESULTS

### The microbiome of healthy Acanthaster cf. solaris tissues.

Healthy CoTS displayed significant tissue differences in their microbiome based on weighted UniFrac distances (permutational multivariate analysis of variance [PERMANOVA]: pseudo-*F*, 10.38, *P* = 0.0001; analysis of similarities [ANOSIM]: *R* = 0.7854, *P* = 0.0001) and individual operational taxonomic units (OTUs) (PERMANOVA: pseudo-*F*, 5.30, *P* = 0.0001; ANOSIM: *R* = 0.7037, *P* = 0.0001). More specifically, the male gonad microbiome differed from all other tissues based on individual OTUs (PERMANOVA and ANOSIM, *P* < 0.05).

**FIG 2:**
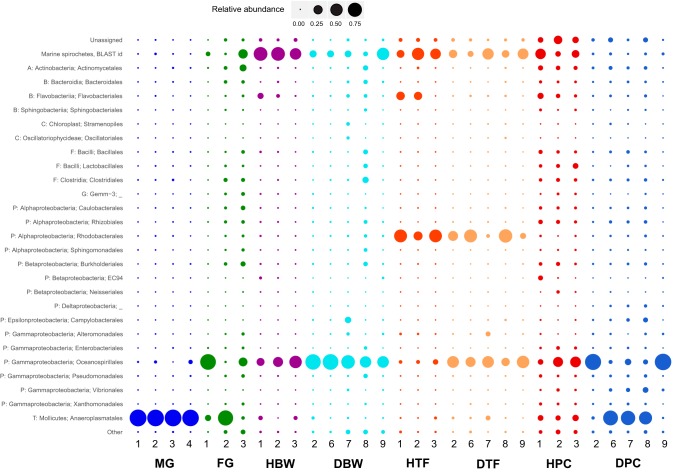
Taxonomic composition of amplicon sequences from healthy Acanthaster cf. solaris tissue samples. Labels reflect the phylum (abbreviated), class, and order as assigned by the QIIME pipeline. OTUs that could not be assigned to a taxonomic group by the QIIME pipeline are categorized as unassigned, with the exception of two OTUs (Unassigned_OTU1 and Unassigned_OTU2) categorized as marine spirochetes based on their best BLAST matches, as discussed in the text. Orders with relative abundances of >1% in at least one sample are shown, with remaining taxa included in the category “other.” Abbreviations: A, Actinobacteria; B, Bacteroidetes; C, Cyanobacteria; F, Firmicutes: G, Gemmatimonadetes; P, Proteobacteria; T, Tenericutes; MG, male gonads; FG, female gonads; HBW, healthy body wall; HTF, healthy tube feet; HPC, healthy pyloric ceca. The associated number identifies the sampled individual as described in Table S5 in the supplemental material.

**FIG 3:**
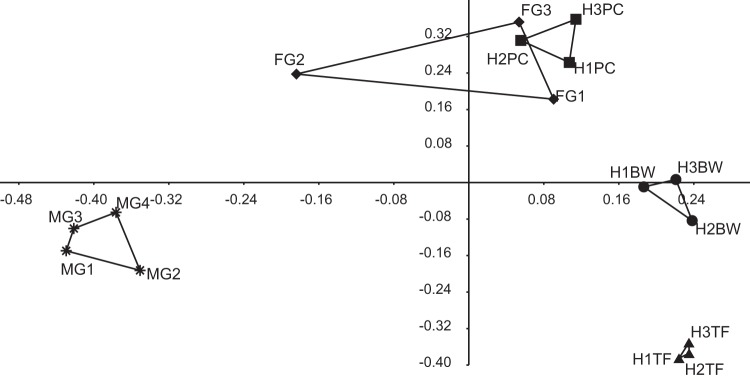
Principal-coordinate analysis (PCoA) plot based on Bray-Curtis similarities of Hellinger (square root)-transformed OTU abundance data evenly subsampled to 7,824 reads. Abbreviations: HBW, healthy body wall; HTF, healthy tube feet; HPC, healthy pyloric ceca; FG, female gonads; MG, male gonads. The number in the sample label identifies the sampled individual as described in Table S5 in the supplemental material.

The male gonad microbiome was dominated by a single OTU, classified by QIIME to the order Anaeroplasmatales (Anaeroplasmataceae_OTU1; 96.0 to 99.6% of reads) (Fig. 2; see also Fig. S1 in the supplemental material). This dominance of a single OTU resulted in a tight cluster in principal-component analysis (PCoA) plots for male gonad tissue samples (Fig. 3 and S2), a high dominance value (Fig. S3), and low values for evenness (Shannon), species richness (observed species), Fisher's alpha, and overall phylogenetic distance (PD whole tree) (Fig. S3). The same OTU was detected in all healthy tissue samples, albeit at lower relative abundances (pyloric ceca, 2.7 to 7.7%; female gonads, 0.2 to 79.8%; tube feet, ≤0.1%; body wall, <0.1 to 3.4%) (Fig. S1 and S4). This single OTU was significantly associated with male gonads and explained 9.0% of the dissimilarity between healthy tissues overall (Table S1). In particular, it explained large proportions of the dissimilarity between male gonads and tube feet (22.1%) or body wall (19.1%) and also between male gonads and female gonads (11.8%) or pyloric ceca (8.3%) (Table S1). The only other order detected in male gonads at an average relative abundance of >1% was Oceanospirillales (0.0 to 3.5%) (Fig. 2).

**FIG 4:**
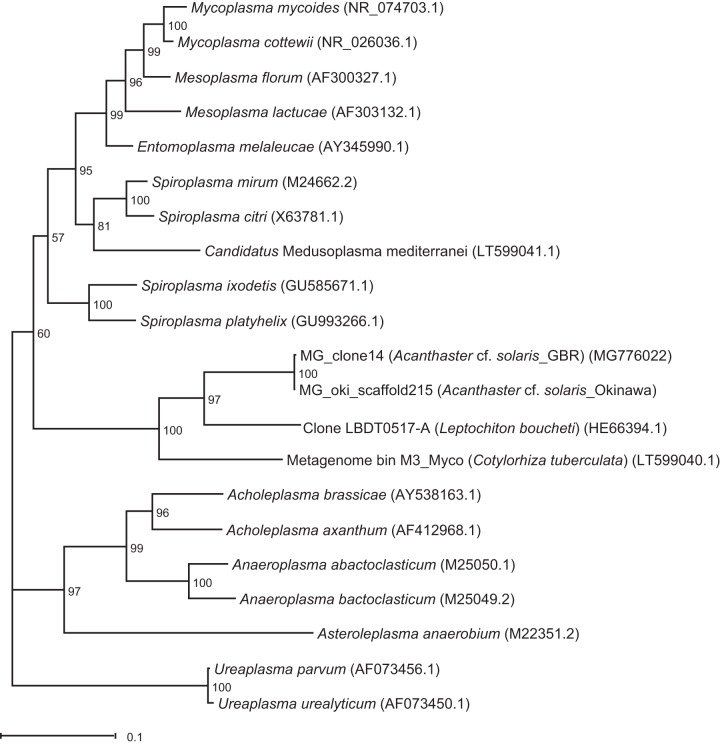
Maximum likelihood tree showing the phylogenetic position within the Mollicutes of the dominant bacterium in Acanthaster cf. solaris male gonads. The sequence MG_clone14 was cloned from male gonads of *Acanthaster* cf. *solaris* collected from the Great Barrier Reef. The sequence MG_ oki_scaffold215 was extracted from an existing scaffold produced from male gonads of Acanthaster cf. solaris collected near Okinawa (5). Bootstrap values are based on 1,000 bootstrap replications. The scale bar represents the number of substitutions per site. GenBank accession numbers are given in parentheses.

**FIG 5:**
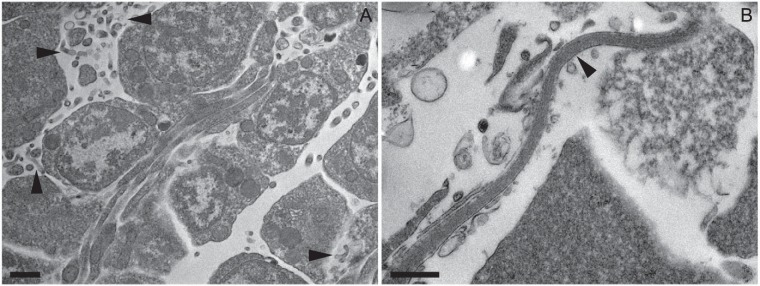
Transmission electron micrographs from healthy Acanthaster cf. solaris tissues. (A) The spermatogenic layer of a male gonad showing bacterial morphologies (arrowheads) similar to those of Spiroplasma in exponential growth and its pleiomorphic or intermediate forms. Scale bar, 1 μm. (B) A spirochete-shaped bacterium (arrowhead) detected in the coelomic epithelium of tube feet. Scale bar, 500 nm.

The phylogenetic position of the dominant OTU in male gonads was analyzed in greater detail. Nine 16S rRNA gene clones derived from male gonads were Sanger sequenced and found to have 99.7 to 100% sequence identity across the analyzed 1,495 bases. A representative clone had 99.6% identity, including two single base deletions present in all clones, to a 16S rRNA gene sequence recovered from a scaffold previously generated for male gonads from a CoTS collected near Okinawa, Japan (5). The closest sequence matches in the nonredundant nucleotide (nr/nt) database were two uncultured Mollicutes clones from the chiton Leptochiton boucheti (NCBI accession number HE663394; 85% sequence identity) (30) and from the jellyfish Cotylorhiza tuberculata (LT599040; 83% sequence identity) (31). The closest matches in the 16S rRNA database were Spiroplasma platyhelix (NCBI accession number GU993266; 80% sequence identity) (32) and Spiroplasma ixodetis (GU585671; 81% sequence identity) (33). These results were supported by the generated phylogenetic tree (Fig. 4). The sequences derived from CoTS male gonads (GBR and Okinawa) clustered closely together, with the chiton-derived sequence as the closest relative. The cluster formed a deep branch with the Spiroplasma-derived lineages, which include the Spiroplasma clades (Citri-Chrysopicola-Mirum, Apis, and Ixodetis) and the Mycoides-Entomoplasmataceae clade (34) (Fig. 4). Transmission electron microscopy (TEM) of male gonads detected cells compatible with both helical and pleiomorphic or intermediate forms of Spiroplasma in the spermatogenic layer (Fig. 5A), linking the dominant retrieved bacterial sequences to the characteristic morphologies of this taxon (35).

**FIG 6:**
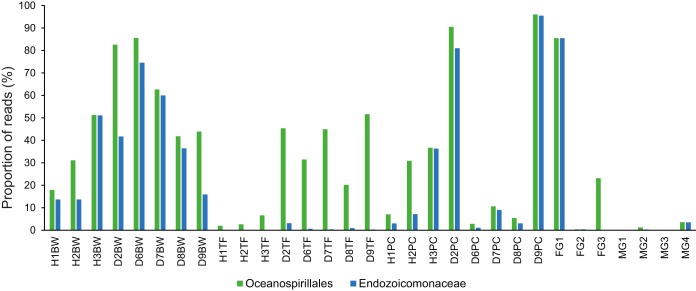
Proportion of reads classified as Oceanospirillales and Endozoicomonaceae by QIIME for healthy and diseased Acanthaster cf. solaris tissue samples. Abbreviations: H, healthy; D, diseased; BW, body wall; TF, tube feet; PC, pyloric ceca. The number in the sample label identifies the sampled individual as described in Table S5 in the supplemental material.

Female gonads displayed large variation in their microbiomes with the relative abundances of Oceanospirillales- and Anaeroplasmatales-related sequences in particular differing among individuals (Fig. 2). One sample was dominated by the order Oceanospirillales (85.5%) (Fig. 2), of which nearly all reads (>99.9%) were classified as belonging to Endozoicomonaceae (genus Endozoicomonas, family Hahellaceae) (Fig. 6). Another sample had high relative abundance of Anaeroplasmatales-related sequences (79.9%) (Fig. 2 and S1), driving this sample toward the male gonad samples in PCoA plots (Fig. 3 and S2). One OTU related to Caulobacterales was significantly associated with female gonads even though it explained <2% of the dissimilarity between female gonads and other individual tissues (Table S1).

Body wall samples from healthy individuals had a high relative abundance (45.1 to 65.8%) of unassigned reads, largely belonging to two OTUs (Unassigned_OTU1, 38.6 to 61.7%; Unassigned_OTU2, 1.5 to 12.5%) (Fig. S1). BLAST searches for representative sequences showed that these two OTUs are related to spirochetes previously detected in marine invertebrates (Table S2). Hence, they were grouped and labeled “marine spirochetes, BLAST id” in Fig. 2 to distinguish them from the other unassigned OTUs shown. Unassigned_OTU1 was significantly associated with body wall samples (Table S1). It explained relatively large proportions of the dissimilarity between body wall and female and male gonads (8.5% and 14.4% of the dissimilarity, respectively) (Table S1). Both of the marine spirochete-related OTUs were detected in all healthy and diseased somatic tissue samples except that Unassigned_OTU2 was absent from one diseased tube foot sample. Hence, our results suggest that marine spirochetes are part of a core CoTS microbiome (Table S3).

The order Oceanospirillales accounted for 17.9 to 51.3% of reads from body wall samples (Fig. 2 and 6), and of those 44.0 to 99.7% were Endozoicomonas (Fig. 6). Three Endozoicomonas-related OTUs (Endozoicomonaceae_OTU1, Endozoicomonaceae_OTU2, and Endozoicomonaceae_OTU3) together explained relatively large proportions of the dissimilarity between body wall and other individual tissues; however, no individual Endozoicomonas-related OTU was significantly associated with the body wall (Table S1). Endozoicomonaceae_OTU1 was detected in all healthy and diseased somatic tissues, and Endozoicomonaceae_OTU2 and Endozoicomonaceae_OTU3 were detected in all healthy and diseased body wall and pyloric cecal samples; hence, they are likely members of a core CoTS microbiome (Table S3). Furthermore, three additional Endozoicomonas-related OTUs were present in all healthy and all diseased body wall samples at low relative abundances (Table S3) (Endozoicomonaceae_OTU5, Endozoicomonaceae_OTU6, and Endozoicomonaceae_OTU7; up to 0.2% each). Only three other taxa were detected in healthy body wall samples at an average relative abundance of >1% in at least one individual, namely, Flavobacteriales (0.1 to 9.2%), Anaeroplasmatales (<0.1 to 3.9%), and the betaproteobacterial order EC94 (<0.1 to 1.0%) (Fig. 2).

Tube foot samples from healthy individuals had high relative abundance of the order Rhodobacterales (24.2 to 55.3%) (Fig. 3), with nearly all (99.9%) classified to family level as Hyphomonadaceae. The Hyphomonadaceae-related OTU was significantly associated with tube feet and explained 13.5%, 6.3%, 8.1%, and 15.1% of the dissimilarity between tube feet and body wall, pyloric ceca, female gonads, and male gonads, respectively (Table S1). This OTU was present in all healthy and all diseased tube foot samples (Fig. S1 and Table S3). A large proportion (up to 52.2%) of reads from healthy tube feet were unassigned, with the majority (86.5 to 94.1%) belonging to Unassigned_OTU1, tentatively identified as a marine spirochete, as described above. Interestingly, a spirochete-shaped cell was evident in the coelomic epithelium of the tube foot wall (Fig. 5B). Two additional unassigned OTUs (Unassigned_OTU4 and Unassigned_OTU5) were present in all healthy and diseased tube foot samples (Table S3) and significantly associated with tube feet, despite having low relative abundance (up to 0.7% each) and explaining <2% of the overall dissimilarity between tissue groups (Table S1). BLAST searches for representative sequences indicated that Unassigned_OTU4 was related to Hyphomonadaceae, while Unassigned_OTU5 had very low sequence identity (<90%) with sequences in public databases, with the closest cultured relatives belonging to the phylum Firmicutes (Table S2). Another three unassigned OTUs (Unassigned_OTU, Unassigned_OTU8, and Unassigned_OTU9) were detected in all tube foot samples irrespective of health status (Table S3), albeit at low relative abundances (up to 0.4%). Only three additional orders were present in healthy tube feet at an average relative abundance of >1% in at least one individual: Flavobacteriales (0.1 to 21.3%), Oceanospirillales (2.0 to 6.6%), and Anaeroplasmatales (<0.1 to 1.0%) (Fig. 2). An OTU related to Flavobacterium explained between 2.5% and 6.7% of the dissimilarity between tube feet and other tissues; however, the association was not significant due to large variability between individuals (Fig. S1). The proportion of Oceanospirillales reads identified as belonging to the Endozoicomonas was low in all healthy tube foot samples (1.7 to 9.3%) (Fig. 6). Of the six Oceanospirillales-related OTUs that were detected in all healthy and diseased tube feet, only two were classified as Endozoicomonas (Table S3).

Pyloric ceca of healthy individuals had microbiomes with relatively high alpha diversity (Fig. S3). This was reflected in a high number of orders with average read abundances above 1% (Fig. 2) and the highest proportion (3.3 to 4.7%) of reads assigned to orders with relative abundances of <1% each (Fig. 2, Other). Unassigned reads constituted up to 41.8%, with 25.0 to 82.8% of these belonging to the OTUs tentatively identified by BLAST as spirochetes (Unassigned_OTU1 and Unassigned_OTU2) (Fig. 2). A third unassigned OTU (Unassigned_OTU3) also present in all healthy and diseased pyloric ceca (Table S3) was tentatively identified by BLAST as an epsilonproteobacterium (Table S2) and significantly associated with pyloric ceca (Table S1). The relative read abundance of Oceanospirillales and proportion of Endozoicomonas were in the ranges of 7.0 to 36.7% and 23.2 to 99.0%, respectively (Fig. 2 and 6), with individuals following the same trend as for the corresponding body wall samples (Fig. 6). All three Oceanospirillales-related OTUs that were detected in all healthy and diseased pyloric ceca belonged to Endozoicomonas (Table S3). Another Endozoicomonas-related OTU (Endozoicomonaceae*_*OTU4) was significantly associated with pyloric ceca but explained <2% of the dissimilarity with other tissues (Table S1). Other orders with relative abundances above 1% in pyloric ceca were Anaeroplasmatales (6.0 to 10.3%), Flavobacteriales (1.3 to 9.4%), Lactobacillales (1.8 to 7.6%), Actinomycetales (2.9 to 5.6%), Rhizobiales (1.3 to 3.9%), Bacillales (1.1 to 3.1%), Burkholderiales (0.7 to 3.0%), Clostridiales (1.7 to 2.6%), Enterobacterales (0.5 to 2.2%), Pseudomonadales (0.6 to 1.6%), Neisseriales (<0.1 to 1.6%), Vibrionales (0.3 to 1.5%), Caulobacterales (1.0 to 1.5%), Bacteroidales (0.7 to 1.3%), and Xanthomonadales (0.5 to 1.2%) (Fig. 2). Individual OTUs related to Anaeroplasmatales, Bacillales, Caulobacterales, and Vibrionales were detected in all pyloric cecal samples (Table S3), and OTUs related to Actinomycetales, Bacillales, Lactobacillales, Rhizobiales, Burkholderiales, Enterobacterales, and Vibrionales were significantly associated with pyloric ceca even though each explained <2% of the overall dissimilarity between healthy tissue samples (Table S1).

### Comparative analyses of healthy and diseased tissues.

**FIG 7:**
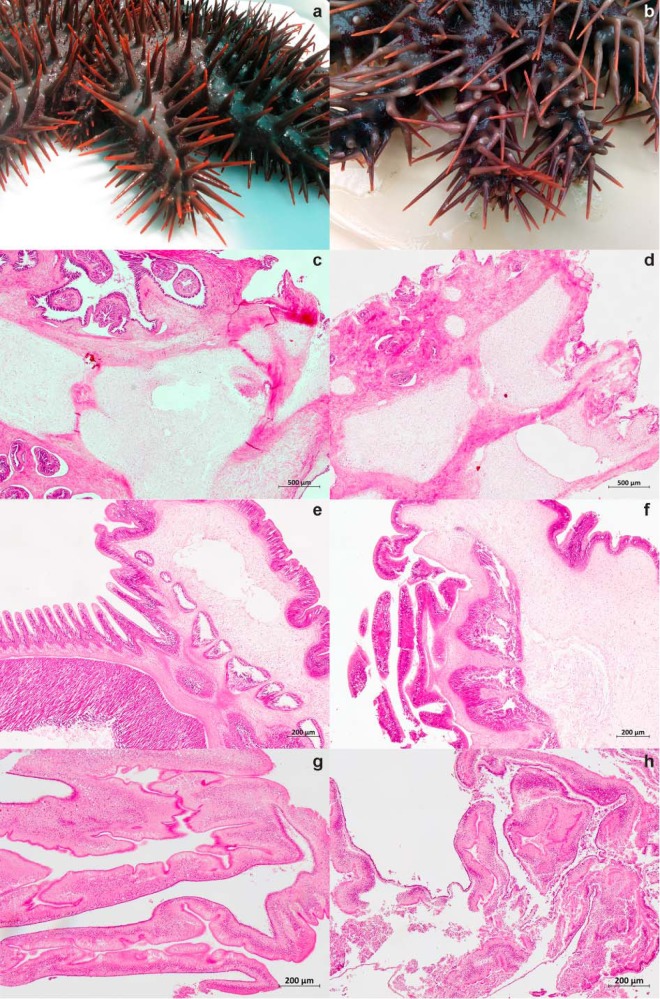
Photos and micrographs showing representative healthy and diseased Acanthaster cf. solaris samples. The micrographs were produced by automated tiling and stitching as indicated. (a and b) Arms of healthy (a) and diseased (b) individuals. (c to h) The following hematoxylin- and eosin-stained tissue sections are also shown: body wall from healthy (c) and diseased (d) individuals (5-by-5 tiles, 10× objective; scale bar, 500 μm); tube feet from healthy (e) and diseased (f) individuals (5-by-5 tiles; 20× objective; scale bar, 200 μm); pyloric ceca from healthy (g) and diseased (h) individuals (4-by-4 tiles; 20× objective; scale bar, 200 μm).

Histological analysis revealed tissue disintegration in diseased individuals. Transverse sections of body wall showed reduced tissue integrity, with papulae frequently replaced by voids (Fig. 7). The structural integrity of tube feet was largely retained in diseased individuals; however, in some cases the integument was loosening, and the nonadhesive epidermis was disrupted. The structural integrity of pyloric ceca was clearly affected. The extent of damage ranged from nearly intact areas with few changes, via loosening of the tunica serosa and the underlying nervous layer and muscle fibers, to more severe disintegration.

Microbiome 16S rRNA gene profiling of healthy and diseased somatic tissues showed that both tissue and health status explained significant parts of the variation based on phylogenetic distance (two-way PERMANOVA; *P* = 0.0001 and *P* = 0.0002) and individual OTUs (two-way PERMANOVA; *P* = 0.0001 and *P* = 0.0126). There was no significant interaction between the two explanatory variables (two-way PERMANOVA; *P* > 0.05). There was a significant increase in dominance for diseased relative to healthy pyloric ceca, and while not significant, there was a general trend of a decrease in all other diversity measures for this tissue type (Fig. S3). In contrast, the opposite trends were seen for diseased relative to healthy body walls (Fig. S3). For tube feet, there were minimal changes in diversity measures between healthy and diseased individuals (Fig. S3).

Increased relative abundance of Oceanospirillales- and Endozoicomonas-related OTUs together explained more than 12.5% of the dissimilarity between healthy and diseased individuals (Table S4). In particular, there was a clear increase in the relative abundance of Oceanospirillales in diseased tube feet (Fig. 2 and 6), mostly due to two OTUs (Oceanospirillales*_*OTU1 and Oceanospirillales*_*OTU3) closely related to the type strain of Kistimonas asteriae (Fig. S1 and Table S2). While these OTUs were present in all healthy and all diseased tube feet (Fig. S1 and Table S3), Oceanospirillales*_*OTU1 was significantly associated with diseased individuals and explained 3.9% and 12.8% of the dissimilarity between healthy and diseased tissues overall and between healthy and diseased tube feet, respectively (Table S4). Oceanospirillales*_*OTU3 was also significantly associated with diseased tube feet and explained a further 2.9% of the dissimilarity of healthy and diseased tube feet (Table S4).

An OTU related to the genus Arcobacterium (class Epsilonproteobacteria, order Campylobacterales) was significantly associated with diseased individuals (Table S4) but explained <2% of the overall dissimilarity between healthy and diseased individuals (Table S4). This OTU was exclusively detected in diseased CoTS; however, it was not present in all diseased individuals (Fig. S1). Due to the well-recognized role of Vibrio spp. as primary and opportunistic pathogens in marine systems, OTUs classified as Vibrionaceae were analyzed separately (Fig. S5). While the true diversity of this family is underestimated by the low resolution of the amplified 16S rRNA gene fragment, we did observe statistically significant trends in some diversity indices. The species richness (observed species) and phylogenetic distance (PD whole tree) of Vibrionaceae-related OTUs were significantly higher in diseased than in healthy individuals. More specifically, species richness and Fisher alpha diversity of Vibrionaceae were significantly higher in diseased than in healthy pyloric ceca (Van der Waerden's *post hoc* test, *P* < 0.05).

Three OTUs were significantly associated with healthy tissues overall. Unassigned_OTU1, tentatively identified as a marine spirochete, was significantly associated with healthy individuals and specifically with healthy body wall and healthy pyloric ceca (Table S4). A Flavobacterium-related OTU explained 2.9% of the overall dissimilarity (Table S4) between healthy and diseased tissues although its presence varied between individuals (Fig. S1). Unassigned_OTU6, which was tentatively identified by BLAST searches as belonging to the phylum Bacteroidetes (Table S2), was significantly associated with healthy tissues despite explaining <2% of the overall dissimilarity (Table S4). Several additional OTUs were found to be significantly associated with healthy pyloric ceca: Unassigned_OTU2 and OTUs related to Streptococcus, Rhizobium, and Enterobacteriaceae (Table S4).

## DISCUSSION

### Microbiomes of healthy Acanthaster cf. solaris tissues.

Microbiome analysis of the ecologically important crown-of-thorns sea star revealed tissue-specific microbial consortia that were largely conserved among individuals, with the exception of a variable microbial community in female gonads. Male gonads were primarily colonized by bacteria that likely represent a novel species, if not a new genus or family, within the Spiroplasma-derived lineages (34, 36). Closely related sequences have been recovered from male gonads of CoTS from both the GBR and Okinawa, Japan, suggesting the possibility of a host-specific association. The sequence evidence was further supported by the presence of bacterial morphologies consistent with exponentially growing and pleomorphic or intermediate forms of Spiroplasma (35) in the spermatogenic layer of male gonads.

Mollicutes have been detected in several marine and freshwater invertebrates, including bryozoans (37), ascidians (38, 39), chitons (30), shrimp (40–42), crayfish (43), and jellyfish (31, 44). Recently, mollicutes were found to be the dominant bacteria in the coelomic fluid of a low number of the analyzed individuals of Acanthaster amurensis and P. pectinifera (17). The role of mollicutes in marine invertebrates is not yet well understood, but Spiroplasma penaei and Spiroplasma eriocheiris have been implicated in disease of aquaculture produced prawns (41, 45) and crabs (46, 47), respectively. A recently proposed new candidate Spiroplasma genus and species, “Candidatus Medusoplasma mediterranei” gen. nov, sp. nov. (31), was described as an intracellular commensal of the jellyfish Cotylorhiza tuberculata with a predicted anaerobic metabolism. Interestingly, Spiroplasma infection of male gonads in the crayfish Pacifastacus leniusculus appeared to reduce sperm production (43). The occurrence and role of mollicutes in a wide range of insects are better documented, where they have been found to occur both intracellularly and extracellularly and in some cases are implicated in male killings during late embryogenesis and protection of their host against parasites (48). The role of the Spiroplasma-related bacterium in CoTS gonads is unknown but worthy of further exploration, especially in relation to potential biological control.

The observed variation between female gonad samples may be related to differences in the developmental stage of the gonads, which has been shown to strongly influence the microbiome of other invertebrates such as the sea anemone Nematostella vectensis (49). Ovarian transmission has been demonstrated for many symbiotic bacteria including spiroplasmas (50) and Oceanospirillales (51), and the detection of high relative abundances of these known symbiotic taxa suggests this possibility for CoTS.

Healthy somatic tissue samples, in particular body wall and tube foot samples, returned high relative abundances of two OTUs identified via BLAST searches as belonging to the phylum Spirochaetes. Spiral-shaped microorganisms are commonly observed by electron microscopy in the subcuticular region of many echinoderms and are referred to as type 2 SCBs (19, 21). Type 2 SCBs have been previously detected in body wall and tube feet of sea stars, and while they are usually spirals, they can vary in morphology from straight rods to spirals with long wavelengths to tightly kinked spirals with short wavelengths (19). In the present study, a likely spirochete cell was detected by TEM in the coelomic epithelium of the tube foot wall. Spirochetes were not reported in previous molecular analyses of echinoderm subcuticular bacteria (18, 22), but it is important to note that Lawrence and coworkers used proteobacterium-specific primers that would miss the phylum Spirochaetes. Spirochetes are dominant members of the core microbiome of several octocorals including the red coral Corallium rubrum (52) and the soft coral Lobophytum pauciflorum (53). They are suggested to play a role in host nutrition and possibly microbial community structuring via production of antimicrobials (52, 53). A low representation of Alphaproteobacteria in the Acanthaster cf. solaris body wall contrasts with previous studies of echinoderm subcuticular bacteria, which have suggested that Alphaproteobacteria are relatively abundant and may play important functional roles in sea stars (22), brittle stars (18), and holothurians (22). Oceanospirillales were detected in all healthy and diseased somatic tissue samples and in all female gonad samples. Endozoicomonas spp. are commonly found in a wide range of marine invertebrates, including corals (scleractinian and octocorals), sea anemones, sponges, tunicates, jellyfish, bivalves, snails, and tubeworms as well as fish (54) although they have not previously been reported from echinoderms. Recovered Endozoicomonas sequences had high sequence identity (up to 100%) to sequences retrieved from other marine invertebrates (see Table S2 in the supplemental material). Microscopy-based studies have shown Endozoicomonas to occur as aggregations in host tissues (54). However, recent whole-genome sequencing of several Endozoicomonas strains showed relatively large genomes and the absence of genome reduction, suggesting the existence of a free-living stage (54, 55). In the present study, we were not able to confirm the presence of bacterial aggregates in CoTS body wall, and fluorescence *in situ* hybridization would be required to spatially localize these cells and confirm their identity. Endozoicomonas bacteria have been suggested to have important functional roles in their host related to nutrient acquisition and provision, structuring of the host microbiome, maintaining health, or causing disease (54). Other Oceanospirillales-related OTUs showed high sequence identity (up to 100%) to sequences previously recovered from corals and sponges and from Kistimonas isolated from a wide range of marine invertebrates (Table S2). Interestingly, the genus Kistimonas and the species Kistimonas asteriae were initially described from isolates retrieved from body wall of Asterias amurensis (56), suggesting that Kistimonas may be commonly associated with sea stars.

The order Flavobacteriales (phylum Bacteroidetes) was detected primarily in body wall, tube feet, and pyloric ceca of two out of the three healthy individuals, with low abundance in the third. The best BLAST match for the representative sequence had low sequence identity (88%) with the Flavobacteriaceae genera Actibacter and Namhaeicola (Table S2). Flavobacteriaceae genera have previously been isolated from echinoderms, including Aquimarina from body wall of Asterias amurensis (56) and Bizionia and Olleya from coelomic fluid of the sea urchin Strongylocentrotus pallidus (57). This suggests that Bacteroidetes and, more specifically, Flavobacteriaceae are common in echinoderms although there may be high variability between individuals and in the genera present.

The tube foot microbiome was dominated by a Hyphomonadaceae-related OTU, which was present in all tube foot samples irrespective of health status and was detected at very low abundance in other tissues. The family Hyphomonadaceae (class Alphaproteobacteria, order Rhodobacterales) includes strict aerobic stalked and nonstalked (one genus only) species that divide by binary fission or budding and are capable of living in low-nutrient environments (36). The presence of stalked bacteria in tube feet could not be confirmed by histology or TEM. Related sequences were previously detected in body wall of the temperate sea star Patiriella sp. (Table S2) (22). Tube feet are part of the water vascular system, and trace amounts of fluid could have been trapped inside the lumen of sampled feet. The fluid of the water vascular system is similar to seawater but includes coelomocytes, which mediate cellular immunity in sea stars (58, 59), a little protein, and an elevated potassium ion content (60). It is unknown to what extent fluid in the water vascular system includes bacteria from the surrounding seawater, and future studies should investigate this possibility.

Pyloric ceca had the most diverse microbiome of all Acanthaster cf. solaris tissues, likely reflecting the presence of bacteria capable of enzymatic degradation of a variety of feed items, as well as microenvironments with various conditions. A high number of taxa commonly associated with gastrointestinal tracts of animals were detected, including Actinomycetales, Bacillales, Bacteroidales, Burkholderiales, Clostridiales, Enterobacterales, Flavobacteriales, Lactobacillales, Neisseriales, Pseudomonadales, Rhizobiales, Vibrionales, and Xanthomonadales (61–64).

### Microbiome shifts in diseased individuals.

A microbial dysbiosis (29) was detected in conjunction with declining host health, involving significant shifts in microbial diversity in body wall and pyloric ceca and significant changes in the relative abundances of some OTUs in all tissues. The most abundant marine spirochete (Unassigned_OTU1) and two OTUs related to Bacteroidetes were significantly associated with healthy individuals, emphasizing that these groups are characteristic members of healthy Acanthaster cf. solaris microbiomes. In contrast, one OTU related to Oceanospirillales (Oceanospirillales_OTU1) and one OTU related to Arcobacter (order Campylobacterales) were significantly associated with diseased individuals.

Body wall samples from diseased individuals had decreased dominance (increased evenness) and a significant loss of marine spirochetes. This loss could be a direct result of habitat disintegration; however, even minor necrosis can attract bacteria capable of colonizing and exploiting available nutrients for rapid proliferation, thereby outcompeting symbionts normally present in healthy individuals (65).

Two OTUs (Oceanospirillales_OTU1 and Oceanospirillales_OTU3) related to the genus Kistimonas (family Hahellaceae) were significantly associated with diseased individuals and, in particular, with diseased tube feet. Related bacteria have been identified as pathogens, including Hahella chejuensis, which was identified as the etiological agent of red egg disease in tilapia hatcheries (66), and Endozoicomonas elysicola, which is responsible for epitheliocystis in cobia hatcheries (67). Kistimonas has so far been reported as living in close association with invertebrate hosts (56, 68, 69), and its mode of transmission is largely unknown. In this study, we cannot exclude the possibility that the detected Kistimonas was present in trace amounts of fluid from the water vascular system trapped in the sampled tube feet. Without more detailed information on the localization and physiology of Kistimonas-related bacteria, it is difficult to speculate on their possible role in CoTS health and disease.

Arcobacter was found only in diseased CoTS but did not occur in all diseased individuals, suggesting that the proliferation of Arcobacter may be opportunistic. The order Campylobacterales (Epsilonproteobacteria) and, specifically, the genus Arcobacter were previously found to dominate the gut microbiome of captive-raised sea urchins (Lytechinus variegatus) (14, 15). Arcobacter has also been detected in diseased coral (70, 71) and necrotic and diseased sponges (65, 72). While Arcobacter is linked to gastrointestinal disease and bacteremia in humans and additionally causes disease in rainbow trout (Oncorhynchus mykiss), its pathogenicity and virulence mechanisms are still poorly characterized (73). Importantly, not all species and strains are pathogenic, with some Arcobacter bacteria being opportunistic pathogens or commensals (74).

No single Vibrio-related OTU was associated with diseased tissues in this study, but the diversity of Vibrionaceae increased in pyloric ceca of diseased individuals, suggesting opportunistic proliferation of Vibrio spp. Although there was no evidence that Vibrio spp. caused the disease event described in the present study, it is possible that members of this genus can cause disease symptoms in CoTS under other circumstances.

We note that the dominant taxa Mollicutes and Endozoicomonas in diseased CoTS include many intracellular bacteria or microorganisms known to occur as dense aggregates in host tissues. It is possible that bacterial cells with an intimate association with host cells or protected by a tightly enveloping membrane (67, 75) can be protected against host immune responses (54) or simply be detectable for a longer period of time after the onset of tissue degradation.

Diseased CoTS individuals are rarely encountered in the wild (76); hence, the sampled disease event in captive CoTS represented an opportunity to investigate possible dysbiosis in CoTS tissues. The comparison to healthy CoTS was done using individuals from a separate, healthy batch of CoTS that were acclimatized in the same aquarium system to minimize any bias introduced by transportation and captivity. While it is expected that a severe disease event would be the strongest driver of the observed differences between healthy and diseased tissues, it cannot be excluded that some differences were introduced by using CoTS from a different reef collected 6 weeks later. Several bacterial taxa were present in all analyzed individuals in this study, some of which were tissue specific; other taxa were present in multiple tissues and therefore part of the core microbiome of CoTS. The spatial and temporal stability of bacterial communities in wild CoTS should be targeted in future studies, including analysis of the different species in the Acanthaster species complex.

This study revealed the presence of tissue-specific microbial communities inhabiting gonads, body wall, tube feet, and pyloric ceca of Acanthaster cf. solaris and demonstrated that dysbiosis occurs in conjunction with declining host health. The functional role that symbionts play in maintaining or disturbing CoTS health and controlling CoTS reproduction should now be investigated to ascertain whether these microorganisms represent an Achilles' heel that could be exploited in future CoTS control efforts.

## MATERIALS AND METHODS

### Collection and sampling of sea stars.

Sea stars were collected from the northern section of the Great Barrier Reef between Cairns and Port Douglas, Queensland, Australia, by the Crown-of-Thorns Starfish Control Program Project (see Table S5 in the supplemental material). After collection by scuba divers, Acanthaster cf. solaris specimens were transferred immediately to purpose-built 1,000-liter holding tanks with trays separating individuals and with a continuous flow of seawater via a spray tower as previously described (77). Trays were transferred to a transporter tank (1,000 liters) with static seawater and constant aeration and transported by car for 5 h to the Australian Institute of Marine Science, Townsville, Australia (77). Upon arrival, CoTS were transferred to outdoor tanks (1,000 liters) with flowthrough unfiltered seawater and aeration.

CoTS collected in late March 2014 developed symptoms of disease upon transfer to outdoor tanks, including drooping spines and inability to adhere to the tank wall (Fig. 7). Three diseased individuals were sampled for microbiome analysis within a week (diseased individual 2 [D2], D6, and D7) and a further two diseased individuals were sampled in the two following weeks (D8 and D9). No lesions were visible at the time of sampling, nor did any develop in sea stars remaining in the tank. Four individuals (D2, D6, D7, and D8) were at an advanced stage of disease progression and possessed little coelomic fluid at the time of sampling, while D9 had more coelomic fluid and appeared to be at an earlier stage of disease progression. CoTS collected in May 2014 were used to obtain baseline information on microbiomes present in tissues of apparently healthy CoTS (healthy individual 1 [H1], H2, and H3). These individuals were acclimated in the outdoor tanks for 4 to 6 days before sampling to confirm their health status after transportation and minimize any tank effects relative to the previous batch. From both healthy and diseased animals, body wall, tube feet, and pyloric ceca (digestive gland) samples were obtained (Fig. 1). The selection of tissues was based on the following considerations: (i) body wall, since many echinoderms harbor subcuticular symbionts and since lesions are a commonly reported disease symptom; (ii) pyloric ceca, since many invertebrate diseases are initiated in the digestive system before becoming systemic; (iii) tube feet, since these are in close contact with coelomocytes, relatively easy to sample, and produce good quality DNA. In addition, gonads were included in the study due to their role in animal reproduction. Outside the spawning season, the gonads of Acanthaster cf. solaris are completely regressed. Hence, gonad tissue samples were obtained from apparently healthy animals collected in November 2013 (male gonad sample 1 [MG1] and MG2) and November 2014 (MG3, MG4, female gonad sample 1 [FG1], FG2, and FG3). All tissues were dissected using sterile scalpels and stored according to their respective downstream analysis.

### DNA extraction, PCR amplification, and next-generation sequencing (NGS).

Samples for DNA extraction were preserved in ethanol (AJA214; ThermoFisher Scientific, USA) with the exception of gonads, which were preserved in RNA Later (ThermoFisher Scientific). Samples in ethanol were left at 4°C for 16 h; then ethanol was exchanged, and the sample was transferred to −20°C for storage. Samples in RNA Later were left at 4°C for 16 h before being transferred to −20°C for storage. DNA was extracted using a ZR Tissue and Insect DNA MiniPrep kit (Zymo Research, USA), as per the manufacturer's recommendations. The quantity and quality of extracted DNA were assessed by agarose gel electrophoresis and by spectrophotometry using a NanoDrop 2000 instrument (ThermoScientific).

Bacterial 16S rRNA genes were amplified and sequenced at the Australian Centre for Ecogenomics (University of Queensland, Australia). Amplification was performed using the primer set 803F (TTAGANACCCNNGTAGTC) and 1392wR (ACGGGCGGTGWGTRC). The primers amplify the V5 to V8 regions of Bacteria and Archaea and were selected based on their high coverage. DNA libraries were prepared with an Illumina TruSeq DNA library preparation protocol, followed by Illumina MiSeq 2- by 300-bp sequencing.

### Bioinformatic/statistical analysis of amplicon sequences.

Due to the length of the amplified fragments, only reverse reads were used for subsequent analysis. Sequences were trimmed using PRINSEQ (*pr*eprocessing and *in*formation of *seq*uence data) Lite, version 0.20.4 (78), and mothur, version 1.34.0 (79). Trimmed sequences were exactly 250 bp long with no ambiguities and a maximum of 8 homopolymers, and all windows (window size 4) had an average quality score of at least 15. Trimmed sequences were analyzed using the QIIME pipeline (version 1.9.0) (80) with the Greengenes database (81), version 13_8 (97% similarity) as a reference.

Chimeric sequences were identified using USEARCH, version 6.1 (82), and filtered from the data set (approximately 1% of reads were removed). Open-reference OTU picking was performed in four steps using UCLUST (82), with a prefilter cutoff of 60%. Singletons and OTUs whose representative sequences could not be aligned with PyNAST were removed. OTUs that were present in the negative extraction control at a relative abundance of more than 0.05% were removed from all samples. Taxonomy was assigned to OTUs by UCLUST. In addition, BLAST searches were performed for the representative sequences of selected OTUs (see below).

Before diversity analyses, sequences were evenly subsampled to 7,824 reads per sample (the lowest read number) (Table S6) to remove the effect of sampling effort. The subsampled data set was also used for similarity percentage (SIMPER) analysis (83) and to identify OTUs that were significantly associated with a group (see below). The OTU table was filtered to retain only selected taxonomic groups using the QIIME script filter_taxa_from_otu_table.py and to retain only OTUs detected in all samples in a defined group using the QIIME script compute_core_microbiome.py, as required. Venn diagrams were generated using the R package VennDiagram, version 1.6.19 (84).

Calculated alpha diversity metrics included dominance, Shannon index, observed species, and PD whole tree. Data were tested for normality using the Kolmogorov-Smirnov and Shapiro-Wilk tests, and homogeneity of variances was tested using Levene's test, using PAST, version 3.04 (85). Variances were generally not homogenous, and the number of samples in each group differed; hence, differences between means were analyzed by a nonparametric Van der Waerden normal scores test followed by a Van der Waerden *post hoc* test (86) with *P* values adjusted for multiple comparisons (87), using the R package PMCMR, version 4.1 (88).

Weighted and unweighted UniFrac distance matrixes were generated by QIIME. Principal coordinates analysis (PCoA), ANOSIM, two-way PERMANOVA (9,999 permutations), and SIMPER analysis were performed using PAST, version 3.04 (85). SIMPER analysis was performed on square root-transformed data with the Bray-Curtis similarity measure. The association of each OTU to a particular group of samples was analyzed using the function signassoc in the R package indicspecies (89). The *P* value was corrected for multiple testing using the Sidak method.

OTUs were selected for further analysis if they explained more than 2% of the dissimilarity between groups (SIMPER) and/or fulfilled the following criteria: (i) were identified by the function signassoc to be significantly associated with a group (*P* < 0.05) and (ii) had an arithmetic average difference in relative abundance between groups of >0.05% (90). Representative sequences for selected OTUs were used to search public sequence databases (nr/nt and 16S rRNA sequences [Bacteria and Archaea]) for closely related matches using BLASTn. The significance level was set at 0.05 in all cases.

### Phylogenetic analysis of 16S rRNA gene sequences.

Nearly full-length bacterial 16S rRNA gene sequences corresponding to the dominant OTU in male gonads (Anaeroplasmataceae_OTU1) were obtained from male gonads by cloning and Sanger sequencing. Briefly, bacterial 16S rRNA gene sequences were amplified from DNA extracted as described above using the primers 27F/1492R (91). The amplification product was purified using a QIAquick PCR purification kit (Qiagen, Germany) and cloned using a TOPO TA cloning kit with competent One Shot TOP10 cells (Invitrogen, USA). Plasmid DNA was purified with a QIAprep Spin Miniprep kit (Qiagen) and Sanger sequenced (Macrogen, South Korea) using M13 primers (M13F/M13R-pUC).

The CoTS (submitted as A. planci) genome sequencing project (5) used male gonads as their starting material. Screening of early scaffolds (not filtered for bacterial sequences) identified one scaffold generated from a specimen collected near Okinawa, Japan, that included the representative sequence of Anaeroplasmataceae_OTU1. WebMGA (92) was used to extract the full-length 16S rRNA gene sequence from this scaffold (oki_scaffold215_size448669).

Related sequences in public databases (nr/nt and 16S rRNA [Bacteria and Archaea]) were identified by nucleotide BLAST. Identified sequences and 16S rRNA gene sequences from related type strains were downloaded and used to create a maximum likelihood-based phylogenetic tree (93). CLC Genomics Workbench, version 9.5.3 (Qiagen), was used for sequence alignment, trimming (about 1,400 bp), model testing, and tree construction using the neighbor-joining algorithm for the starting tree, the general time-reversible (GTR) substitution model (94), and 1,000 bootstrap replicates. The resulting tree was exported and edited for clarity using Dendroscope (95) and Adobe Illustrator.

### Histology and transmission electron microscopy.

Samples for histology were fixed in Bouin's fixative for 16 h at 4°C followed by three rinses in 3× phosphate-buffered saline (1× PBS is 10 mM PO_4_^3−^, 137 mM NaCl, and 2.7 mM KCl, pH 7.4) and storage in 70% ethanol at 4°C until processing. Body wall samples were decalcified in 10% formic acid. All samples were embedded in paraffin, and sections (5 μm) were stained by hematoxylin and eosin. Mounted slides were inspected by an AxioImager M2 compound microscope (Carl Zeiss Pty. Ltd., Oberkochen, Germany), and micrographs were captured by an Axiocam 503 (Carl Zeiss) microscope camera. The microscope software Zen Blue (version 2.3) (Carl Zeiss) was used for automated tiling and stitching of images.

Samples for transmission electron microscopy (TEM) were fixed in 2.5% glutaraldehyde–2% paraformaldehyde in 100 mM cacodylate for about 16 h at 4°C, followed by two rinses in 3× PBS, one rinse in 1× PBS, and storage in 1× PBS at 4°C until processing at the Centre for Microscopy, Characterization, and Analysis at the University of Western Australia. Samples were postfixed in 1% OsO_4_ in PBS and dehydrated in a graded series of ethanol and acetone using a microwave (BioWave; PELCO) before being infiltrated and embedded in Procure-Araldite resin. Sections from healthy male gonads and tube feet were subsequently cut at a thickness of 100 nm on a diamond knife before being stained with 1% aqueous uranyl acetate and Sato's modified lead citrate for 5 min each. All sections were imaged at 120 kV in a TEM (JEOL 2100) fitted with a digital camera (Orius; Gatan).

### Accession number(s).

The raw amplicon data were submitted to the NCBI database under BioProject accession number PRJNA420398, SRA accession number SRP128607, and BioSample accession numbers SRX3542029 to SRX3542037. Sequences of 16S rRNA gene clones were submitted to NCBI's GenBank under accession numbers MG776016 to MG776024.

## Supplementary Material

Supplemental material
