# Supplementary material for: Crown-of-Thorns Sea Star Acanthaster cf. solaris Has Tissue-Characteristic Microbiomes with Potential Roles in Health and Reproduction
Source: Appl Environ Microbiol. 2018 Jun 18;84(13):e00181-18. doi: 10.1128/AEM.00181-18 (PMC6007096; doi:10.1128/AEM.00181-18)
Supplement: Supplemental material [file supp_84_13_e00181-18__index.html]

Supplemental material 

# Crown-of-Thorns Sea Star Acanthaster cf. solaris Has Tissue-Characteristic Microbiomes with Potential Roles in Health and Reproduction

## Supplemental material

- Supplemental file 1 -

  Relative abundances of OTUs identified by SIMPER as explaining >2% of the dissimilarity between samples or identified by the function signassoc to be significantly associated with a specific tissue or health state (Fig. S1); PCoA plot for healthy *Acanthaster* cf. *solaris* tissue samples based on weighted Unifrac distances (Fig. S2); alpha diversity measures for healthy and diseased *Acanthaster* cf. *solaris* tissues (Fig. S3); Venn diagram showing the number of unique and shared OTUs present in all replicates of healthy tissue samples (Fig. S4); read numbers of OTUs classified by QIIME as belonging to the family *Vibrionaceae* in the resampled data set (Fig. S5); tissue-characteristic OTUs as determined by SIMPER and signassoc analyses (Table S1); BLAST analysis results for representative sequences of OTUs identified by SIMPER as explaining >2% of the dissimilarity between samples or identified by signassoc to be significantly associated with a tissue or health status (Table S2); OTUs detected in somatic tissues of all healthy and all diseased individuals (Table S3); OTUs characteristic of healthy and diseased tissues as determined by SIMPER and signassoc analyses (Table S4); metadata for individual *Acanthaster* cf. *solaris* starfish and their sampled tissues (Table S5); sequencing statistics (Table S6).

  PDF, 1.5M
