# Supplementary material for: Crown-of-Thorns Sea Star Acanthaster cf. solaris Has Tissue-Characteristic Microbiomes with Potential Roles in Health and Reproduction
Source: Appl Environ Microbiol. 2018 Jun 18;84(13):e00181-18. doi: 10.1128/AEM.00181-18 (PMC6007096; doi:10.1128/AEM.00181-18)
Supplement: Supplemental material [file AEM.00181-18_zam013188596s1.pdf]

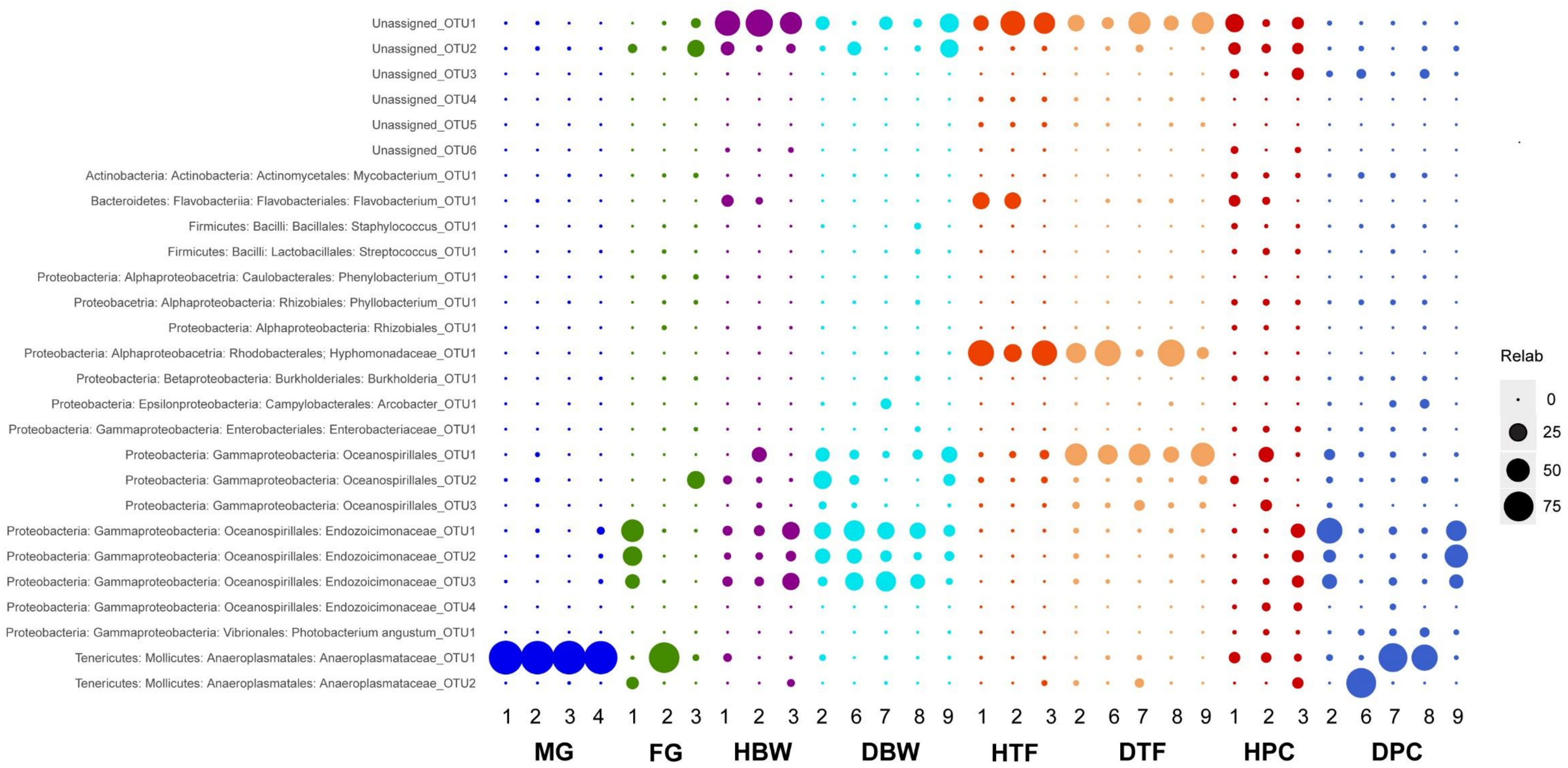

FIG. S1 Relative abundance of OTUs identified by SIMPER as explaining > 2% of the dissimilarity between samples, or identified by the function signassoc to be significantly associated with a specific tissue or health state. The OTU label reflects the taxonomic assignment given by the QIIME pipeline. Abbreviations: MG: male gonads; FG: female gonads; HBW: healthy body wall; DBW: diseased body wall; HTF: healthy tube feet; DTF: diseased tube feet; HPC: healthy pyloric caeca; DPC: diseased pyloric caeca. The number identifies the sampled individual.

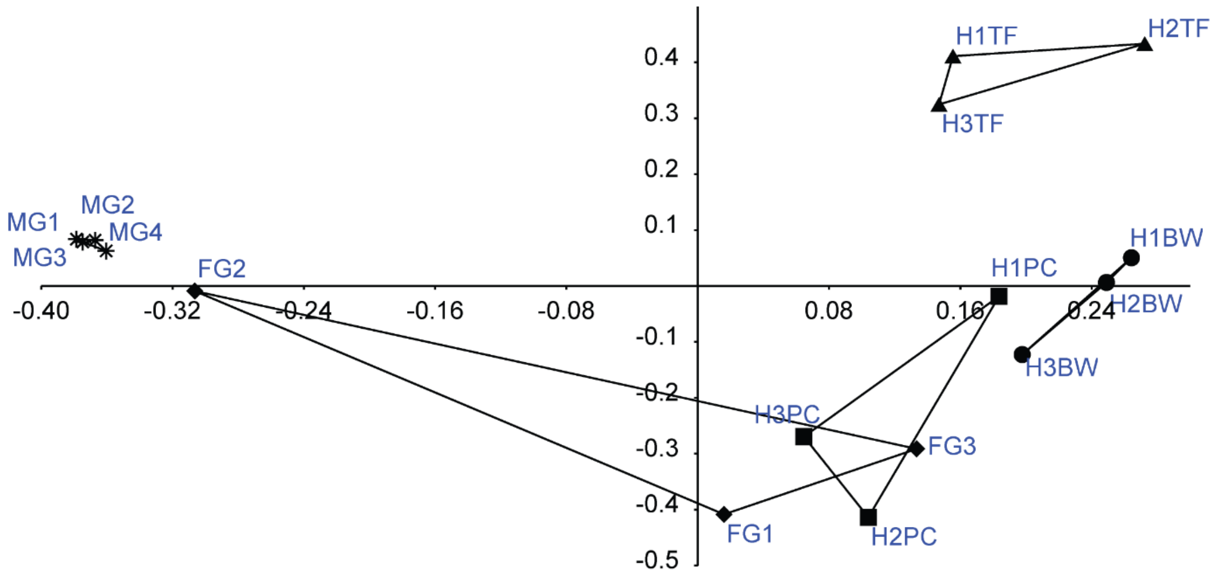

FIG. S2 Principal coordinates analysis (PCoA) plot for healthy *Acanthaster* cf. *solaris* tissue samples based on weighted Unifrac distances. Abbreviations: HBW: healthy body wall; HTF: healthy tube feet; HPC: healthy pyloric caeca; FG: female gonads; MG: male gonads.

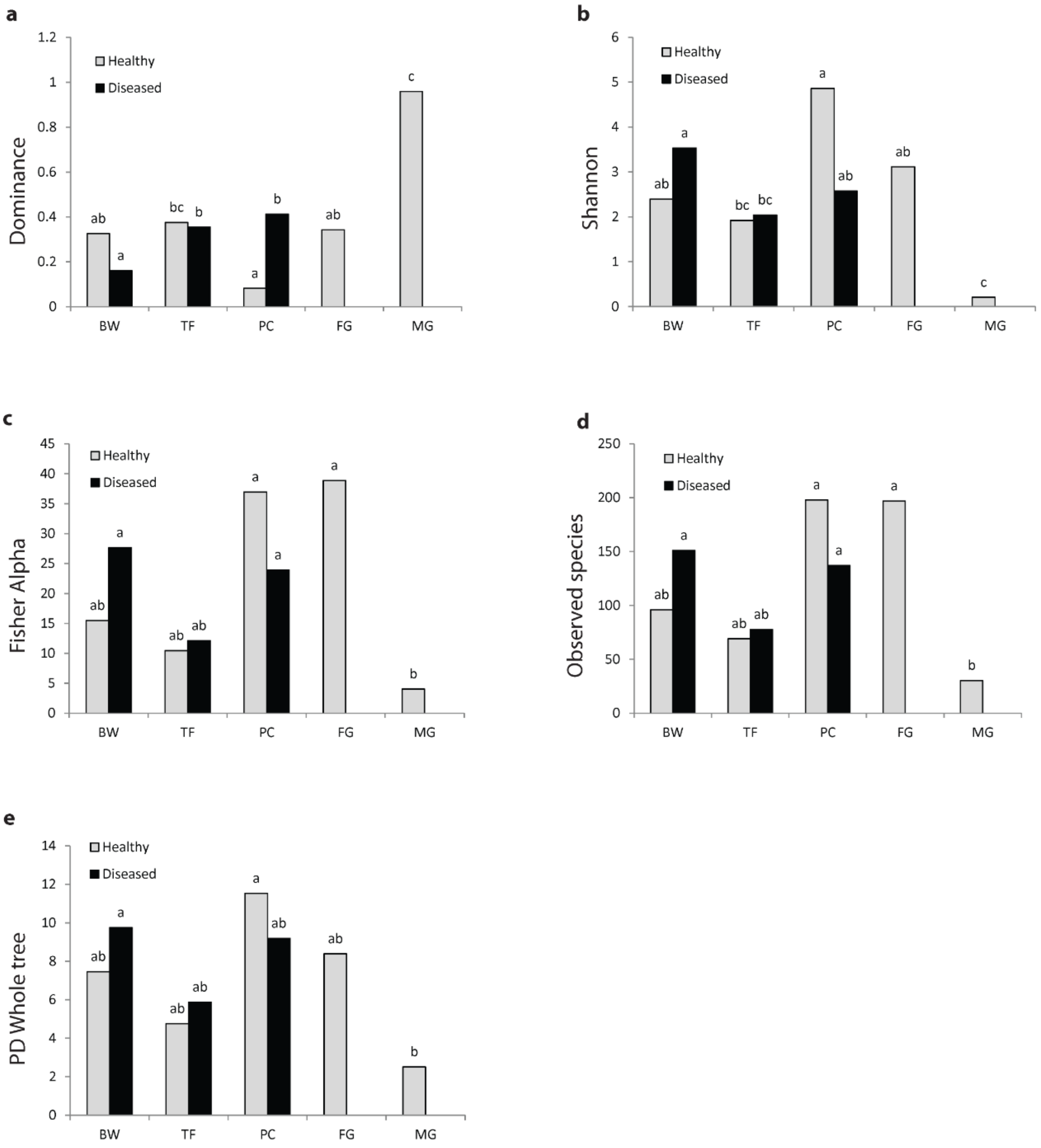

FIG S3 Alpha diversity measures for healthy and diseased *Acanthaster* cf. *solaris* tissues. Letters indicate which samples were statistically different (van der Waal; p<0.05). Abbreviations: BW: body wall; TF: tube feet; PC: pyloric caeca; FG: female gonads; MG: male gonads.

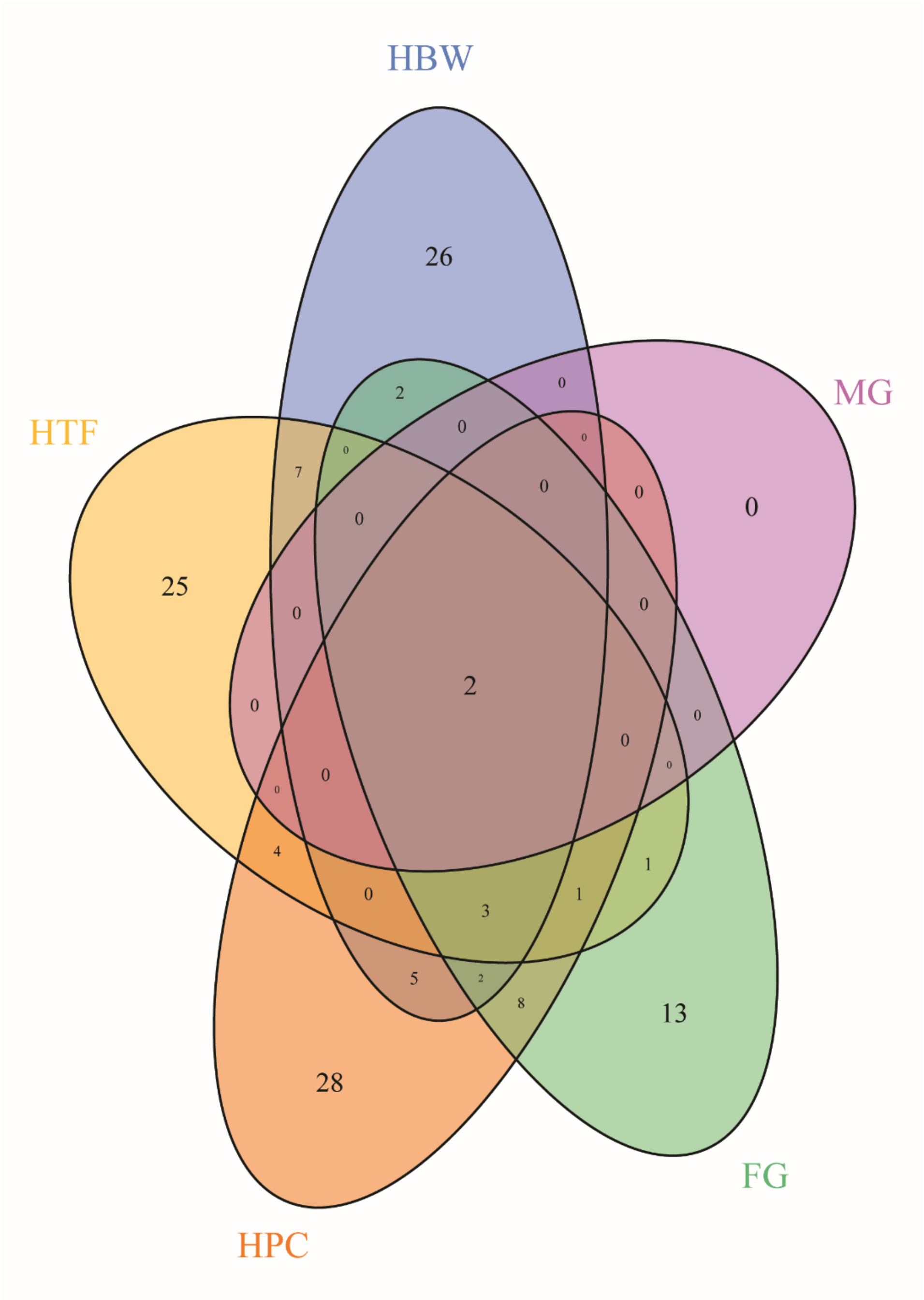

FIG S4 Venn diagram showing the number of unique and shared OTUs present in all replicates of healthy tissue samples. Abbreviations: HBW: healthy body wall (n=3), HTF: healthy tube feet (n=3), HPC: healthy pyloric caeca (n=3), FG: female gonads (n=3), MG: male gonads (n=4).

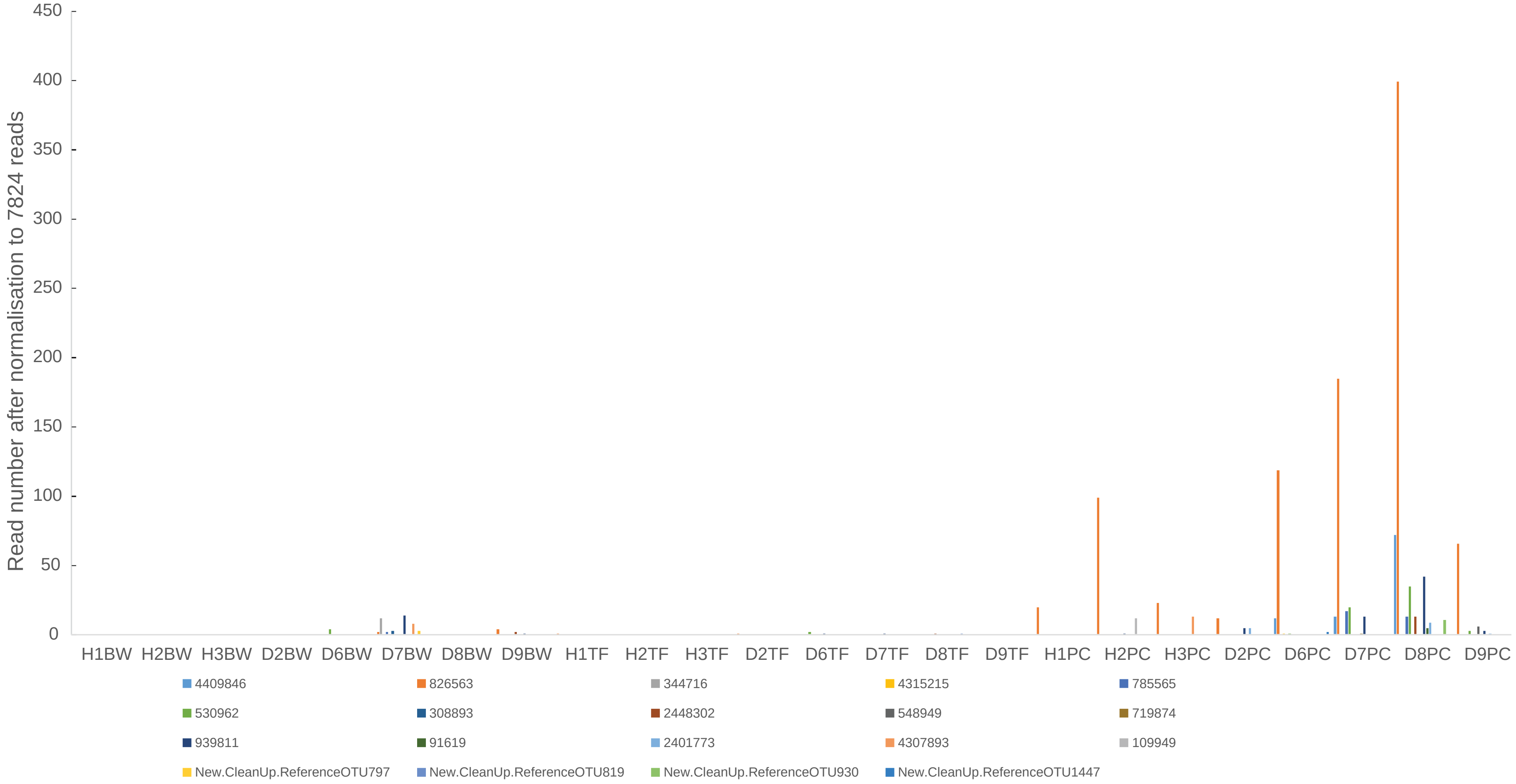

FIG S5 Read numbers of OTUs classified by QIIME as belonging to the family Vibrionaceae in resampled dataset (total reads 7824 per sample).

TABLE S1 Tissue characteristic OTUs as determined by SIMPER and signassoc analyses. OTU labels reflect taxonomic assignment by the QIIME pipeline.

OTU label	All healthy	BW vs TF	BW vs PC	BW vs FG	BW vs MG	TF vs PC	TF vs FG	TF vs MG	PC vs FG	PC vs MG	FG vs MG
Unassigned_OTU1	BW: 6.3%, *	BW: 3.3%, -	BW: 4.2%, -	BW: 8.5%, -	BW: 14.4%, -	TF: 2.7%, -	TF: 6.2%, -	TF: 12.7%, -	PC: 2.3%, -	PC: 3.3%, -	
Unassigned_OTU2	PC: 2.2%, -	BW: 3.4%, -			BW: 4.0%, -	PC: 2.0%, -				PC: 2.5%, -	FG: 3.1%, -
Unassigned_OTU3	PC: <2%, **		PC: 2.0%, -							PC: 2.1%, -	
Unassigned_OTU4	TF: <2%, **										
Unassigned_OTU5	TF: <2%, **										
Actinomycetales: Mycobacterium	PC: <2%, *										
Flavobacteriales: Flavobacterium	TF: 2.8%, -	TF: 5.2%, -			BW: 2.8%, -	TF: 2.5%, -	TF: 3.7%, -	TF: 6.7%, -			
Bacillales: Staphylococcus	PC: <2%, **										
Lactobacillales: Streptococcus	PC: <2%, **										
Caulobacterales: Phenylobacterium	FG: <2%, **										
Rhizobiales: Phyllobacterium	**PC: <2%, ****										
Rhizobiales	**PC: <2%, ***										
Rhodobacterales; Hyphomonadaceae	TF: 4.3%, **	TF: 13.5%, -				TF: 6.3%, -	TF: 8.1%, -	TF: 15.1%			
Burkholderiales: Burkholderia	PC: <2%, **										
Enterobacteriales: Enterobacteriaceae	PC: <2%, **										
Oceanospirillales_OTU1		TF: 3.6%, -			BW: 2.9%, -			TF: 2.9%, -			
Oceanospirillales_OTU2				FG: 2.2%, -							
Oceanospirillales: Endozoicimonaceae_OTU1	BW: 3.4%, -	BW: 6.2%, -	BW: 2.2%, -	BW: 3.9%, -	BW: 5.7%, -		FG: 3.8%, -		FG: 2.5%, -		FG: 5.6%, -
Oceanospirillales: Endozoicimonaceae_OTU2	FG, BW: 2.5%, -	BW: 3.7%, -		BW: 3.5%, -	BW: 3.4%, -		FG: 3.3%, -		FG: 2.3%, -		FG: 4.8%, -
Oceanospirillales: Endozoicimonaceae_OTU3	BW: 2.8%, -	BW: 6.4%, -		BW: 2.8%, -	BW: 6.1%, -		FG: 2.4%, -				FG: 3.4%, -
Oceanospirillales: Endozoicimonaceae_OTU4	PC: <2%, **										
Vibrionales: Photobacterium angustum	PC: <2%, **										
Anaeroplasmatales: Anaeroplasmataceae_OTU1	MG: 9.0%, ***			FG: 3.8%, -	MG: 19.1%, -		FG: 3.4%, -	MG: 22.1%, -	FG: 2.8%, -	MG: 8.3%, -	MG: 11.8%, -
Anaeroplasmatales: Anaeroplasmataceae_OTU2				FG: 2.0%, -							FG: 2.8%, -

Entry format: tissue with highest relative abundance: percentage of dissimilarity explained by SIMPER, significance level in signassoc analysis.

Abbreviations: BW: body wall; TF: tube feet; PC: pyloric caeca; FG: female gonads; MG: male gonads; -: p>0.05; *: p<0.05; **: p<0.01; ***: p<0.005.

TABLE S2 BLAST analysis results for representative sequences of OTUs identified by SIMPER as explaining > 2% of the dissimilarity between samples, or identified by signassoc to be significantly associated with a tissue or health status.

OTU label	QIIME id (id to template)	BLAST nr best matches (acc. no.; seq. id.; host or environment)	BLAST ref_seq best matches (acc. no.; seq. id.; isolation source)
Unassigned_OTU1	Unassigned (84.8%)	Unc. spirochete, isolate SRODG048 (FM995181.1; 86%; oyster *Saccostrea glomerata*)<br><br>Unc. Spirochaetes, clone OTU75 (KP174132.1; 86%; gorgonian *Muricea fruticosa*)	Candidatus *Hepatoplasma crinochetorum* (NR_121778.1; 93%; isopod *Armadillidium vulgare*)<br><br>*Brachyspira intermedia* strain PWS/A (NR_102932.1; 93%; weaned pigs with diarrhea)
Unassigned_OTU2	Unassigned (90.4%)	Unc. Spirochaetes, clone OTU6 (KP174129.1; 93%; zoanthid *Parazoanthus lucificum*)<br><br>Unc. organism clone ctg_CGOAA65TR (DQ395458.1; 92%; deep-sea octocoral)	*Spirochaeta americana* strain ASpG1 (NR_028820.1; 89%; soda lake sediment)<br><br>*Spirochaeta alkalica* strain Z-7491 (NR_026301.1; 88%; cyanobacterial mat in alkaline lake)
Unassigned_OTU3	Unassigned (88%)	Unc. bacterium clone 4132B37 (EU555133.1; 91%; hydrothermal vent)<br><br>Unc. epsilon proteobacterium ML320J-2 (AF458287.1; 91%; alkaline hypersaline lake)	*Nitratiruptor tergarcus* strain MI55-1 (NR_041023.1; 90%; deep-sea hydrothermal system)<br><br>*Campylobacter lari* RM2100 strain RM2100 (NR_0745555.1; 89%; *homo sapiens* clinical isolate)
Unassigned_OTU4	Unassigned (82.8%)	Uncultured alpha proteobacterium clone DPC033 (DQ269093.1; 88%; *Delisea pulchra*)<br>Uncultured bacterium clone denovo7567 (KU637957.1; 88%; red algae *Asparagopsis*)	*Hyphomonas oceanitis* strain SCH-89 (NR_024941.1; 96%; Baltic sea)<br>*Hyphomonas hirschiana* strain VP-5 (NR_024939.1; 96%; Galapagos vent area)
Unassigned_OTU5	Unassigned (81.2%)	Uncultured *Synechococcus* sp. clone JL-WNPG-T40 (AY664136.1; 83%; coastal water)<br>Uncultured bacterium clone denovo31172 (KU629690.1; 89%; red algae *Asparagopsis*)	*Virgibacillus kekensis* strain YIM kkny16 (NR_042744.1; 88%; Salt Lake Keke)<br>*Weissella soli* strain Mi268 (NR_025642.1; 87%; soil)
Unassigned_OTU6	Unassigned (77.6%)	Uncultured bacterium clone LKC09_005_13 (JX521528.1; 91%, terrestrial sulfidic spring)<br>Uncultured bacterium clone (HQ839406.1, 89%, hydrothermal vent)	*Schleiferia thermophila* strain TU-20 (NR_117928.1, 88%, hot spring)<br>*Draconibacterium sediminis* strain MCCC 1A00734 (NR_136493.1, 89%,river sediment)
Actinomycetales; Mycobacterium	*Mycobacterium* (95.2%)	*Mycobacterium abscessus* subsp. *bolletii* strain Y9 (KU861820.1; 100%; tap water)<br>*Mycobacterium chelonae* strain 96-1705 (KT779789.1; 100%, foot biopsy)	*Mycobacterium saopaulense* strain EPM10906 (NR_145859.1; 100%; cornea)<br>*Mycobacterium abscessus* strain ATCC 19977 (NR_114658.1; 100%)
Anaeroplasmatales; Anaeroplasmataceae_OTU1	*Anaeroplasmataceae* (90.8%)	Unc. bacterium, clone LBDT0517-A (HE663394.1; 91%; chiton *Leptochiton boucheti*)<br>Unc. bacterium, clone LBDT7266 (HE663315.1; 91%; chiton *Leptochiton boucheti*)	*Mycoplasma cottewii* strain VIS (NR_026036.1; 85%; goat *Capra hircus*)<br>*Spiroplasma leucomae* strain SMA (NR_104721.1; 89%; satin moth larvae *Stilpnotia salicis*)
Anaeroplasmatales; Anaeroplasmataceae_OTU2	*Anaeroplasmataceae* (90.8%)	Unc. bacterium, clone LBDT0517-A (HE663394.1; 91%; chiton *Leptochiton boucheti*)	*Mycoplasma putrefaciens* Mput9231 (NR_103942.1; 83%; goat *Capra hircus*)

		Unc. bacterium, clone LBDT7266<br>(HE663315.1; 91%; chiton *Leptochiton boucheti*)	*Mycoplasma putrefaciens* KS1<br>(NR_025971.1; 83%; goat *Capra hircus*)
Bacillales; Staphylococcus	*Staphylococcus*<br>(96.4%)	*Lactobacillus plantarum* strain CAU:563 (MF355142.1, 100%, fermented milk)<br>*Staphylococcus epidermidis* strain AS9 (MF286538.1, 100%, mouse skin)	*Staphylococcus epidermidis* strain NBRC 100911 (NR_113957.1, 100%)<br>*Staphylococcus saccharolyticus* strain JCM 1768 (NR_113405.1, 100%)
Burkholderiales; Burkholderia	*Burkholderia*<br>(91.6%)	*Burkholderia* sp. strain BacI48 (KX641532.1, 100%, root tissues)<br>*Burkholderia* sp. symbiont clone (LT221702.1, 100%, stinkbug *Dicranocephalus lateralis* gut)	*Burkholderia peredens* strain LMG 29314 (NR_145606.1, 100%)<br>*Burkholderia turbans* strain LMG 29316 (NR_145604.1, 100%)
Campylobacterales; Arcobacter	*Arcobacter*<br>(86.8%)	*Campylobacteraceae* bacterium FA150<br>(KU143707.1; 99%; sediment)<br>Unc. bacterium clone DLPYS_MD03_095 (KC852778.1; 99%; abscess on scallop *Patinopecten yessoensis*)	*Arcobacter aquimarinus* strain W63<br>(NR_136421.1; 99%; sea water)<br>*Arcobacter cloacae* strain SW28-13<br>(NR_117570.1; 99%; sewage)
Caulobacterales; Phenylobacterium	*Phenylobacterium*<br>(89.2%)	Uncultured bacterium clone UA_79 (JX120449.1, 100%, subsurface aquifer sediment)<br>Uncultured organism (HQ809737.1, 100%, human gastrointestinal specimens)	*Phenylobacterium composti* strain 4T-6 (NR_044377.1, 99%, cotton waste compost)<br>*Phenylobacterium lituiforme* strain FaiI3 (NR_029117.1, 99%, subsurface aquifer)
Enterobacetriales; Enterobacetriaceae	*Enterobacteriaceae*<br>(95.6%)	*Escherichia coli* strain MWW<br>(LC215416.1; 100%; waste water)<br>*Shigella* sp. strain KIB7<br>(KY466922.1; 100%; river water)	*Citrobacter koser*i strain LMG 5519<br>(NR_117751.1; 100%; blood culture)<br>*Trabulsiella odontotermitis* strain Eant 3-9 (NR_043860.1; 100%: termite *Odontotermes formosanus* Shiraki gut)
Flavobacteriales; Flavobacterium	Flavobacterium<br>(84%)	Unc. *Flavobacteriaceae* clone 758_WTL<br>(KP887599.1; 92%; coastal surface waters)<br><br>Unc. *Flavobacteriaceae* clone 0306_MLB20<br>(JQ515052.1; 92%; filtered seawater)	*Actibacter sediminis* JC2129<br>(NR_044349.1; 88%; tidal flat sediment)<br><br>*Namhaeicola litoreus* DPG-25<br>(NR_132282.1; 88%; seawater)
Lactobacillales; Streptococcus	*Streptococcus*<br>(92.4%)	Uncultured bacterium clone OTU0118<br>(KY454989.1; 100%; coral *Acropora hemprichii*)<br>Uncultured bacterium clone denovo3254<br>(KU630575.1; 100%; red algae *Asparagopsis*)	*Streptococcus tigurinus* strain AZ_3a<br>(NR_118234.1; 100%; blood culture)<br>*Streptococcus oralis* strain ATCC 35037<br>(NR_116210.1; 100%; human mouth)
Oceanospirillales_OTU1	*Oceanospirillales*<br>(94%)	Unc. bacterium, clone C430/GW1046<br>(HG423475.1; 98%; sponge *Astrosclera willeyana*)<br><br>Unc. bacterium, clone C467/GW1046<br>(HG423531.1; 98%; sponge *Astrosclera willeyana*)	*Kistimonas asteriae* KMD 001<br>(NR_116386.1; 98%; seastar *Asterias amurensis* skin)<br><br>*Alcanivorax dieselolei* B5<br>(NR_074734.1; 96%; oil-contaminated surface water)
Oceanospirillales_OTU2	*Oceanospirillales*<br>(92%)	Unc. bacterium clone JI4_16<br>(EU487857.1; 100%; lucinid *Phacoides pectinatus*)<br><br>Unc. bacterium clone JI4_12<br>(EU487853.1; 97%; lucinid *Phacoides pectinatus*)	*Kistimonas scapharcae* A36<br>(NR_109422.1; 97%); dead ark clam *Scapharca broughtonii*)<br><br>*Kistimonas asteriae* KMD 001<br>(NR_116386.1; 95%; seastar *Asterias amurensis* skin)

Oceanospirillales_OTU3	*Oceanospirillales* (92.8%)	*Kistimonas asteriae* KMD 001 (NR_116386.1; 100%; seastar *Asterias amurensis* skin)<br><br>*Kistimonas* sp. 1804 (KF444399.1; 99%; toxic Ribbon worm *Cephalothrix simula*)	*Kistimonas asteriae* KMD 001 (NR_116386.1; 100%; seastar *Asterias amurensis* skin)<br><br>*Kistimonas scapharcae* A36 (NR_109422.1; 98%; ark clam *Scapharca broughtonii*)
Oceanospirillales; Endozoicimonaceae_OTU1	*Endozoicimonaceae* (92.4%)	Unc. bacterium clone Apal_H17 (GU118072.1; 100%; coral *Acropora palmata*)<br><br>Unc. bacterium clone Apal_O13 (GU118076.1; 99%; coral *Acropora palmata*)	*Endozoicomonas atrinae* WP70 (NR_134024.1; 97%; comb pen shell *Atrina pectinata*)<br><br>*Endozoicomonas elysicola* MKT110 (NR_041264.1; 96%; sea slug *Elysia ornata*)
Oceanospirillales; Endozoicimonaceae_OTU2	*Endozoicimonaceae* (92.8%)	Unc. bacterium clone 1C227608 (EU799933.1; 100%; seawater)<br><br>Unc. bacterium clone Woods-Hole_a4129 (KF799373.1; 98%; tunicate *Ciona intestinalis*)	*Endozoicomonas atrinae* WP70 (NR_134024.1; 97%; comb pen shell *Atrina pectinata*)<br><br>*Endozoicomonas elysicola* MKT110 (NR_041264.1; 96%; sea slug *Elysia ornata*)
Oceanospirillales; Endozoicimonaceae_OTU3	*Endozoicimonaceae* (88.4%)	Unc. bacterium clone AT88 (FJ809513.1; 99%; coral *Acropora* sp.)<br><br>Unc. bacterium clone AT70 (FJ809495.1; 99%; coral *Acropora* sp.)	*Endozoicomonas euniceicola* EF212 (NR_109684.2; 93%; octocoral *Eunicea fusca*)<br><br>*Endozoicomonas elysicola* MKT110 (NR_041264.1; 92%; sea slug *Elysia ornata*)
Oceanospirillales; Endozoicimonaceae_OTU4	*Endozoicimonaceae* (91.6%)	Unc. gamma proteobacterium clone C23 (DQ884170.1; 100%; ascidian *Cystodytes dellechiajei*)<br><br>Unc. gamma proteobacterium clone C19 (DQ884169.1; 95%; ascidian *Cystodytes dellechiajei*)	*Endozoicomonas numazuensis* HC50 (NR_114318.1; 95%; sponge *Haplosclerida* gen. et sp.)<br><br>*Endozoicomonas elysicola* MKT110 (NR_041264.1; 96%; sea slug *Elysia ornata*)
Rhizobiales	*Rhizobiales* (87.6%)	Uncultured *Xanthobacteraceae* bacterium clone 3.86P (KP235996.1, 96%, soil)<br>Uncultured *Xanthobacteraceae* bacterium clone 3.107P (KP235987.1, 96%, soil)	*Devosia enhydra* (NR_104723.1, 93%, junction between ocean and freshwater lake)<br>*Xanthobacter flavus* strain NBRC 14759 (NR_113665.1, 93%)
Rhizobiales; Phyllobacterium	*Phyllobacterium* (93.2%)	Unc. bacterium clone 6h18 (HG531829.1; 100%; SHIME_human in vitro gut model)<br>Unc. *Phyllobacterium* sp. clone H12 (KF003163.1; 100%; grass carp *Ctenopharyngodon idellus* gut mucus)	*Phyllobacterium ifriqiyense* strain STM 370 (NR_043192.1; 100%; root nodule of *Lathyrus numidicus*)<br>*Phyllobacterium myrsinacearum* strain STM 948 (NR_043189.1; 100%; leaf nodule of *Ardisia crispa*)
Rhodobacterales; Hyphomonadaceae	*Hyphomonadaceae* (89.2%)	Unc. bacterium clone PSE5 (FJ393578.1; 96%; seastar *Patiriella* sp.)<br><br>Unc. bacterium clone PSE34 (FJ393607.1; 95%; seastar *Patiriella* sp.)	*Hirschia maritima* GSW-2 (NR_116900.1; 92%; seawater)<br><br>*Hirschia baltica* ATCC 49814 (NR_074121.1; 92%; brackish fjord water)
Vibrionales; Photobacterium angustum	*Photobacterium angustum* (97.6%)	*Vibrio* sp. strain Vcl_050 (KX279494.1; 100%; coral *Cladocora caespitose*)<br>*Vibrio hyugaensis* strain 090810a (NR_145569.1; 100%; seawater)	*Vibrio alginolyticus* strain NBRC 15630 (NR_122060.1; 100%)<br>*Vibrio parahaemolyticus* strain ATCC 17802 (NR_118569.1; 100%)

Table S3: OTUs detected in somatic tissues of all healthy (n=3) and all diseased (n=5) individuals (total n=8).

	Body wall	Tube feet	Pyloric caeca
Unassigned_OTU1	Y	Y	Y
Unassigned_OTU2	Y		Y
Unassigned_OTU3			Y
Unassigned_OTU4		Y	
Unassigned_OTU5		Y	
Unassigned_OTU7		Y	
Unassigned_OTU8		Y	
Unassigned_OTU9		Y	
Anaeroplasmatales: Anaeroplasmataceae_OTU1 (f)	Y		Y
Bacillales: Staphylococcus (g)			Y
Caulobacterales: Caulobacteraceae (f)			Y
Oceanospirillales_OTU1 (o)		Y	
Oceanospirillales_OTU2 (o)		Y	
Oceanospirillales_OTU3 (o)		Y	
Oceanospirillales_OTU4 (o)		Y	
Oceanospirillales: Endozoicimonaceae_OTU1 (f)	Y	Y	Y
Oceanospirillales: Endozoicimonaceae_OTU2 (f)	Y		Y
Oceanospirillales: Endozoicimonaceae_OTU3 (f)	Y		Y
Oceanospirillales: Endozoicimonaceae_OTU5 (f)	Y		
Oceanospirillales: Endozoicimonaceae_OTU6 (f)	Y		
Oceanospirillales: Endozoicimonaceae_OTU7 (f)	Y		
Oceanospirillales: Endozoicimonaceae_OTU8 (f)		Y	
Rhodobacterales; Hyphomonadaceae (f)		Y	
Vibrionales: Photobacterium angustum (s)			Y

TABLE S4 OTUs characteristic of healthy and diseased tissues as determined by SIMPER and signassoc analyses. The OTU label reflects the taxonomic assignment given by the QIIME pipeline.

**OTU label**	**Health status overall**	**HBW vs DBW**	**HTF vs DTF**	**HPC vs DPC**
Unassigned_OTU1	H: 4.8%, *	HBW: 7.0%, *	HTF: 4.7%, -	HPC: 2.7%, *
Unassigned_OTU2	H: 2.0%, -	DBW: 2.8%, -		HPC: 2.0%, *
Unassigned_OTU6	H: <2%, ***			
Anaeroplasmatales: Anaeroplasmataceae_OTU1	D: 2.2%, -			DPC: 3.0%, -
Anaeroplasmatales: Anaeroplasmataceae_OTU2			DTF: 2.1%, -	DPC: 2.3%, -
Campylobacterales: Arcobacter	D: <2%, **			
Enterobacteriales: Enterobacteriaceae				HPC: <2%, *
Flavobacteriales: Flavobacterium	H: 2.9%, ***	HBW: 2.4%, -	HTF: 8.4%, -	
Lactobacillales: Streptococcus				HPC: <2%, *
Rhizobiales				HPC: <2%, *
Rhodobacterales: Hyphomonadaceae	H: 4.6%, -		HTF: 7.3%, -	
Oceanospirillales_OTU1	D: 3.9%, *	DBW: 3.8%, -	DTF: 12.8%, *	
Oceanospirillales_OTU2		DBW: 3.1%, -		
Oceanospirillales_OTU3			DTF: 2.9%, *	
Oceanospirillales: Endozoicimonaceae_OTU1	D: 3.4%, -	DBW: 2.7%, -		DPC: 2.7%, -
Oceanospirillales: Endozoicimonaceae_OTU2	D: 2.5%, -	DBW: 2.3%, -		DPC: 2.1%, -
Oceanospirillales: Endozoicimonaceae_OTU3	D: 2.7%, -	DBW: 2.7%, -		

Entry format: health status with highest relative abundance: percentage of dissimilarity explained by SIMPER, significance level in signassoc analysis.

Abbreviations: H: healthy; D: diseased; HBW: healthy body wall; DBW: diseased body wall; HTF: healthy tube feet; DTF: diseased tube feet; HPC: healthy pyloric caeca; DPC: diseased pyloric caeca;

-: $p>0.05$; *: $p<0.05$; **: $p<0.01$; ***: $p<0.005$.

Table S5: Metadata for individual *Acanthaster* cf *solaris* and their sampled tissues.

Collection Date	Reef*	Individuals	Health status	Sampling Date	Tissues	Sample labels
30.10.2013	Arlington Reef	MG1, MG2	Healthy	12.11.2013	Gonad (testis)	MG1, MG2
26.03.2014	Lark Reef	D2	Diseased	23.04.2014	Body wall, Tube feet, Pyloric caeca	D2BW, D2TF, D2PC
26.03.2014	Lark Reef	D6	Diseased	29.04.2014	Body wall, Tube feet, Pyloric caeca	D6BW, D6TF, D6PC
26.03.2014	Lark Reef	D7	Diseased	30.04.2014	Body wall, Tube feet, Pyloric caeca	D7BW, D7TF, D7PC
26.03.2014	Lark Reef	D8	Diseased	06.05.2014	Body wall, Tube feet, Pyloric caeca	D8BW, D8TF, D8PC
26.03.2014	Lark Reef	D9	Diseased	20.05.2014	Body wall, Tube feet, Pyloric caeca	D9BW, D9TF, D9PC
07.05.2014	Mackay Reef	H1	Healthy	12.05.2014	Body wall, Tube feet, Pyloric caeca	H1BW, H1TF, H1PC
07.05.2014	Mackay Reef	H2	Healthy	13.05.2014	Body wall, Tube feet, Pyloric caeca	H2BW, H2TF, H2PC
07.05.2014	Mackay Reef	H3	Healthy	14.05.2014	Body wall, Tube feet, Pyloric caeca	H3BW, H3TF, H3PC
24.09.2014	Rudder Reef	MG3, MG4	Healthy	17.11.2014	Gonad (testis)	MG3, MG4
24.09.2014	Rudder Reef	FG1, FG2, FG3	Healthy	17.11.2014	Gonad (ovary)	FG1, FG2, FG3

*All reefs are located in the Cooktoown/Lizard Island and Cairns sectors of the GB

TABLE S6 Sequencing statistics. The filtering step included removal of chimeras, singletons, sequences that could not be aligned with PyNAST, and sequences assigned to OTUs detected in an extraction control at relative abundance > 0.05%.

Sample	Total reads	Reads after qc	Reads after filtering
H1BW	65884	48847	47049
H2BW	55391	42355	41282
H3BW	71088	51860	50301
D2BW	38725	28354	27391
D6BW	52766	37833	36305
D7BW	64360	44824	43270
D8BW	17145	12847	7824
D9BW	50356	15413	14920
H1TF	77807	57181	55026
H2TF	72644	56020	54446
H3TF	79524	58648	57156
D2TF	84236	62958	61152
D6TF	92124	67841	65392
D7TF	69584	49729	48419
D8TF	85258	63951	62581
D9TF	69777	22207	21421
H1PC	61834	25403	7852
H2PC	99246	48559	17548
H3PC	79071	34979	14398
D2PC	85491	41000	28737
D6PC	80404	32799	13496
D7PC	62362	24889	10289
D8PC	52773	29440	13693
D9PC	85496	21570	18255
FG1	69267	49013	45396
FG2	118694	38632	22945
FG3	93797	37881	11006
MG1	116187	67313	66553
MG2	76677	34530	33464
MG3	65305	52694	49921
MG4	73609	55536	53543

Abbreviations: H: healthy; D: diseased; BW: body wall; TF: tube feet; PC: pyloric caeca; FG: female gonads; MG: male gonads. Numbers refer to the sampled CoTS individual.
